# Association of clinical symptoms and cardiometabolic dysregulations in patients with schizophrenia spectrum disorders

**DOI:** 10.1192/j.eurpsy.2023.2477

**Published:** 2023-12-13

**Authors:** Chenxu Zhao, Tesfa Dejenie Habtewold, Elnaz Naderi, Edith J. Liemburg, GROUP Investigators, Richard Bruggeman, Behrooz Z. Alizadeh

**Affiliations:** 1 Department of Epidemiology, University Medical Center Groningen, University of Groningen, Groningen, The Netherlands; 2 Department of Psychiatry, Rob Giel Research Center, University Center for Psychiatry, Groningen, The Netherlands

**Keywords:** cardiometabolic biomarkers, cognition, metabolic syndrome, psychotic symptoms, schizophrenia

## Abstract

**Background:**

Patients with schizophrenia spectrum disorders (SSD) have a shortened life expectancy related to cardiovascular diseases. We investigated the association of cognitive, positive, and negative symptoms with cardiometabolic dysregulations in SSD patients.

**Methods:**

Overall, 1,119 patients from the Genetic Risk and Outcome in Psychosis (GROUP) study were included. Cognitive function, positive and negative symptoms were assessed at baseline, 3-year, and 6-year. Cardiometabolic biomarkers were measured at 3-year follow-up. We used linear and multinomial logistic regression models to test the association between cardiometabolic biomarkers and clinical trajectories and performed mediation analyzes, while adjusting for clinical and demographic confounders.

**Results:**

Cognitive performance was inversely associated with increased body mass index (mean difference [β], β_high_ = −1.24, 95% CI = –2.28 to 0.20, *P* = 0.02) and systolic blood pressure (β_mild_ = 2.74, 95% CI = 0.11 to 5.37, *P* = 0.04). The severity of positive symptoms was associated with increased glycated hemoglobin (HbA1c) levels (β_low_ = −2.01, 95% CI = −3.21 to −0.82, *P* = 0.001). Increased diastolic blood pressure (OR_high-decreased_ = 1.04, 95% CI = 1.01 to 1.08, *P* = 0.02; OR_high-increased_ = 1.04, 95% CI = 1.00 to 1.08, *P* = 0.048) and decreased high-density lipoprotein (OR _high-increased_ = 6.25, 95% CI = 1.81 to 21.59, *P* = 0.004) were associated with more severe negative symptoms. Increased HbA1c (OR_moderate_ = 1.05, 95% CI = 1.01 to 1.10, *P* = 0.024; OR_high_ = 1.08, 95% CI = 1.02 to 1.14, *P* = 0.006) was associated with more severe positive symptoms. These associations were not mediated by antipsychotics.

**Conclusions:**

We showed an association between cardiometabolic dysregulations and clinical and cognitive symptoms in SSD patients. The observed associations underscore the need for early identification of patients at risk of cardiometabolic outcomes.

## Introduction

Schizophrenia spectrum disorder (SSD) is a severe and disabling psychotic disorder manifested by positive symptoms (e.g., delusions, hallucinations, disorganized thinking, and speech), negative symptoms (e.g., social withdrawal, loss of motivation, and reduced communication), and cognitive symptoms (e.g., deficits in attention, concentration, and memory) [1]. Patients with SSD have a 15–20 years shortened life expectancy compared with the general population [2, 3], mostly attributable to the increased risk of cardiovascular diseases [4, 5].

Cardiometabolic disorders such as obesity, type 2 diabetes, and metabolic syndrome (MetS) are the primary risk factors of cardiovascular diseases and compelling causes of shorter life [6]. The specific measurable cardiometabolic biomarkers including body mass index, blood pressure, and cholesterol levels, are closely associated with the presence and development of cardiometabolic disorders. Despite the denouncing effect of cardiometabolic biomarkers on SSD clinical symptoms, the nature of association between them remains to be elucidated. So far, cardiometabolic biomarkers have been related to cognitive impairment, and positive and negative symptoms [7–9] in patients with SSD. High blood glucose and blood pressure are associated with delayed memory, vigilance, processing speed [10–14], and reasoning abilities [15]. Low-density lipoprotein and triglycerides are related to psychotic symptoms, impaired executive function [16], and verbal memory [17]. Impaired cognitive capacities, on the other hand, may increase the risk of developing cardiometabolic disorders subsequently through unhealthy lifestyles including poor diet, less physical activity, and substance abuse [18, 19]. Negative symptoms also impact the cardiometabolic biomarkers due to the lack of autonomous motivation to maintain healthy lifestyles [20].

Genetic liability, side effects of atypical antipsychotics, and inadequate health-care services, may provoke the increasing cardiometabolic disorders in SSD [3]. However, previous results have also suggested that patients with SSD may be genetically predisposed to cardiometabolic disorders independent of antipsychotic side effects [21] as abnormal glucose and lipid metabolism have been observed in drug-naïve SSD patients. Meanwhile, several studies have shown a wide range of results. For instance, systolic blood pressure and glucose level were not correlated with cognitive impairment in Chinese patients with SSD found by Peng et al. [22]. Similarly, Depp et al. [23] reported no association between hypertension and obesity with cognitive ability.

The inconsistent findings are partly caused by methodological differences among studies, inclusion of patients at different stages of the illness, applied statistical modeling, selection of confounders, and misclassifications [10]. Additionally, neglecting the effect of disease heterogeneity could also be a reason causing the discrepancy [24]. In patients experiencing various levels of cognitive deficits, it is unclear whether all patients or only subgroups of patients are at a higher risk of developing cardiometabolic dysregulations. The associations, and absence of associations, found in previous studies need to be validated in a large sample. The mediated effect of the use of antipsychotics has not been tested in previous studies.

The relationship between clinical symptoms and cardiometabolic dysregulation is complex and multifaceted. Rates of non-treatment of cardiometabolic disorders ranged from 30.2% to 88.0% in patients with SSD [25]. Therefore, understanding these associations is crucial for comprehensive management of mental and physical health in patients with SSD. We investigated the relationships between longitudinal cognitive, positive, and negative symptoms trajectories with cardiometabolic biomarkers in patients with SSD from the Dutch national Genetic Risk and Outcome of Psychoses (GROUP) cohort [26]. We also tested whether and how much these possible associations are mediated by antipsychotics.

## Methods

### Study design and participants

This study included 1,119 patients with SSD at baseline from the GROUP cohort study, a multicenter longitudinal study in the Dutch population. Patients were included based on the following criteria: i) age range of 16 to 50 years at baseline (extremes included); ii) a diagnosis of non‐affective psychotic disorder according to the Diagnostic and Statistical Manual of Mental Disorders, Fourth Edition (DSM‐IV) criteria [27]; iii) good command of the Dutch language; and iv) able and willing to give written informed consent. In general, data was collected at enrolment and follow-up measurements approximately at 3-year and 6-year. The blood samples for metabolic biomarkers assay were collected at the 3-year follow-up. All patients from the GROUP study who had measurements for predictors and outcomes were included in the current study. The study protocol was centrally approved by the Ethical Review Board of the University Medical Center Utrecht and by local review boards of each participating institute. Details regarding sample characteristics, recruitment, and assessment procedures have been published elsewhere [26]. GROUP release number 8 was used for the current analyzes.

### Measurements

#### Demographic and clinical characteristics

Patients were asked about demographic information, such as age, gender, ethnicity, education, number of cigarettes use per day, and number of alcohol units use per week. Clinical data were also collected through medical record review.

#### Cardiometabolic biomarkers

All eligible patients at the 3-year follow-up had physical examination and blood assay for biomarkers. Weight (kg), height (m), waist circumference (cm), and systolic (SBP) (mmHg) and diastolic blood pressure (DBP) (mmHg) data were collected by physical examination. Glycated hemoglobin (HbA1c) (mmol/mol), low-density lipoprotein (LDL) (mmol/l) cholesterol, high-density lipoprotein (HDL) (mmol/l) cholesterol, and triglycerides (TG) (mmol/l) levels were measured in the whole blood sample.

MetS was defined using the U.S. National Cholesterol Education Programme Adult Treatment Panel III (NCEP-ATP-III) [28], when any three of the following five features are present: i) waist circumference ≥ 88 cm in women or 102 cm in men; ii) BP ≥130/85 mmHg or being prescribed antihypertensives; iii) HDL cholesterol <50 mg/dl (=1.30 mmol/l) in women or <40 mg/dl (=1.03 mmol/l) in men; or being prescribed HDL increasing drugs; iv) triglycerides ≥150 mg/dl (=1.7 mmol/l) or being prescribed triglyceride-lowering drugs; and v) fasting plasma glucose ≥100 mg/dl [29] (=5.6 mmol/l) or being prescribed antidiabetics. As plasma glucose levels were not available, a HbA1c ≥5.1% (=32 mmol/mol) was used as a criterion [30].

Metabolic composite scores were calculated based on the definition of MetS by summing up the standardized value of each of the components [31]. Each individual mean blood pressure was standardized using mean arterial pressure (MAP). Means and standard deviations of the patients ranging within healthy reference values were used to standardize HDL (≥1.30 mmol/l in female and ≥ 1.03 mmol/l in male patients), TG (<1.7 mmol/l), and HbA1c (<32 mmol/mol). The HDL score was reversed because higher scores represent a better outcome. Finally, the average metabolic composite score was calculated by dividing the sum of all standardized components by five [31] and treated as a continuous value.

#### Cognitive function, positive and negative symptoms

Cognitive function was assessed using the consensus cognitive battery test called “Measurement and Treatment Research to Improve Cognition in Schizophrenia (MATRICS)” [32]. The eight assessment protocols included CPT performance and CPT variance of continuous performance test (CPT-HQ) [33], immediate and delayed recall of word learning task (WLT), and the digit symbol coding, block design, arithmetic, and information subtests of the Wechsler Adult Intelligence Scale (WAIS) III [34, 35]. A shortened version of WAIS III [36] that consists of digit symbol coding subset, and every second (or third) item of block design, information, and the arithmetic was administered at wave 3. At each wave of assessment, the tests used were administered in a fixed order approximately for 2 h with break in case of subject fatigue. Positive and negative symptoms were assessed using the Positive and Negative Syndrome Scale (PANSS) which consists of 30 items with a seven-point Likert scale that characterizes positive and negative symptoms and general psychopathology in patients [37].

#### Clinical trajectories

The clinical trajectory refers to the discernible pattern, such as stability, fluctuations, decline, or improvement, in the overall cognitive functioning and the severity of positive and negative symptoms. To ascertain groups of patients exhibiting similar patterns of cognitive function, as well as levels of positive and negative symptoms, we employed group-based trajectory modeling (GBTM) as described in detail elsewhere [38–40]. This analysis utilized composite scores from cognitive assessments and mean scores of PANSS positive and negative subscales, based on measurements collected at three time points of baseline, 3 years, and 6 years during the follow-up period spanning 6 years. Our previous studies have delineated five distinct cognitive trajectory groups, three distinct positive symptom trajectory groups, and three distinct negative symptom trajectory groups [38–40].

#### Antipsychotics use

We used the type of first prescribed antipsychotics at three waves and sorted as a categorical variable based on the risk of cardiometabolic side effects [41] as the following: i) low risk/no risk: typical antipsychotics, including haloperidol, flupentixol, penfluridol, pimozide, zuclopentixol, broomleridol, perfenazine, and pipamperon; ii) medium risk: atypical antipsychotics without metabolic side effect including aripiprazol, amisulpride, sulpiride, paliperidon, and risperidone; and iii) high risk: atypical antipsychotics with metabolic side effect including olanzapine, quetiapine, and clozapine. The missing data at 3-year and 6-year were imputed using best guest assumption by adjacent wave given the observations that the use of the antipsychotic remained stable in patients over the follow-up period.

### Data analysis

#### Association analysis

One-way ANOVA and Kruskal-Wallis tests were conducted to compare the differences of cardiometabolic biomarkers among clinical trajectories when the cardiometabolic outcomes were normally distributed and not normally distributed, respectively. Post hoc analyzes were followed for pairwise comparisons by *t*-test and Dunn test and adjusted by Bonferroni correction method.

Linear regression models were fitted to regress each of the numerical cardiometabolic components as an outcome over clinical trajectories adjusted for age, gender, education level, ethnicity, IQ, illness duration, cigarette use, and alcohol. Multinomial logistic regression models were fitted using MetS and its components as exposure and clinical trajectories as an outcome adjusted for the above-mentioned confounders as well. A stepwise method was conducted to select the most important candidate predictor variables, with an entry-threshold set as 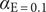 and remove-threshold set as 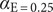 . Metabolic composite score was not modeled in multivariable multinomial logistic regressions to avoid overfitting given that composite score was calculated using the individual metabolic components.

#### Mediation analysis

The cross-sectional mediation analysis [42] of antipsychotics use (mediator) was performed using PROCESS macro [43] between the association of cognitive trajectory (main predictor), and each of nine metabolic components, as well as the metabolic composite score (outcome). The significant predictors of age, sex, ethnicity, education, illness duration, IQ, number of cigarettes use per day, and number of alcohol units use per week in regression results were included in the mediation models. In total, four regression models were fitted: model I on the association of predictors and mediators, model II on the association of predictors and outcome (direct effect), model III on the association of mediator and outcome (indirect effect), and model IV on the association of predictors and outcome without considering mediator. The significance level of direct and total effect was set to α = 0.05, whereas the significance of indirect effect was evaluated by non-parametric bootstrapping with 5,000 bootstrap samples. The indirect effect was determined whether the 95% confidence interval of coefficient estimated by bootstrapping contained zero. The reported effects and direct effect were unstandardized according to the recommendation from Hayes guidelines [44].

#### Sensitivity analysis

We performed sensitivity analyzes to assess the impact of outliers (deviated from mean ± 3 standard deviation) of each cardiometabolic biomarker.

#### Power calculation

For the association of clinical trajectories and cardiometabolic components, the required sample size was 44 observations calculated using G*Power software (version 3.1). We met the requirements (*N* = 1,119), with medium effect size *f*^2^ = 0.25 at α = 0.05, and power of 0.8 with 11 predictors. For the cross-sectional mediation analysis, we used simulation analysis method recommended by Fritz and Mackinnon [45], the required sample size was 78 observations with medium effect size (0.39) in both path A and path B, using percentile bootstrapping method.

## Results

### Demographic and clinical profiles at 3-year follow up

More than three-fourths (76.14%) of patients were male and the mean (±SD) age and age onset of SSD was 30.60 (±7.22) years and 23.07 (±7.81) years, respectively. Patients had an average duration of illness of 8.45 ± 4.44 years at wave 2. In the past 3 years, 41.18% of the patients had more than one psychotic episode. The majority (78.87%) of the patients were currently using antipsychotics. The most used antipsychotics were risperidone (17.82%), olanzapine (23.70), and clozapine (21.80%).

Of the 1,119 patients, 41.64%, 14.39%, and 2.32% had mild, moderate, and severe cognitive impairment, respectively. Similarly, 8.40% and 11.71% of patients had severe positive and negative symptoms, respectively. Detailed characteristics of clinical trajectories could be found in previous papers [46].

Mean (±SD) body mass index (BMI) was 26.11 (±4.87) kg/m^2^. Mean (±SD) TG, HDL cholesterol, and LDL cholesterol were 1.81 (±1.44), 1.24 (±0.63), 3.11 (±0.93) mmol/mol, respectively. Mean estimated DBP and SBP were 79.38 ± 11.04, 127.27 ± 15.28 mmHg, respectively (Table 1).

**Table 1.** Descriptive characteristics of the sample of patients with SSD

Demographic characteristics	
Age, mean years (SD)	30.60 (7.22)
Sex, male *n* (%)	852 (76.14)
Ethnicity, Caucasian *n* (%)	859 (79.24)
Years of education, mean (SD)	12.41 (3.81)
Marital status, *n* (%)	
Not married	930 (85.40)
Married/living together	128 (11.75)
Other (divorced and widowed)	18 (2.85)
Estimated IQ, mean (SD)	98.82 (16.61)
Age onset illness, mean (SD)	23.07 (7.81)
Duration of illness, mean (SD)	8.45 (4.44)
Number of psychotic episodes in the past 3 years *n* (%)	
More than 1 episode	334 (41.18)
No episode	477 (58.82)
Use of antipsychotics *n* (%)	
Not currently using	37 (5.11)
Currently using	571 (78.87)
Unknown if currently using	116 (16.02)
Type of 1st prescribed antipsychotics at wave 2	
Olanzapine (Zyprexa)	137 (23.70)
Clozapine (Leponex)	126 (21.80)
Risperidone (Risperdal)	103 (17.82)
Aripiprazol (Abilify)	71 (12.28)
Quetiapine (Seroquel)	61 (10.55)
Haloperidol (Haldol)	23 (3.98)
Others	57 (9.86)
Cardiometabolic biomarkers, mean (SD)	
Body mass index (kg/m^2^)	26.11 (4.87)
Waist circumference (cm)	95.00 (14.39)
Glycated hemoglobin (mmol/mol)	35.06 (5.87)
Triglycerides (mmol/l)	1.81 (1.44)
High-density lipoprotein (mmol/l)	1.24 (0.63)
Low-density lipoprotein (mmol/l)	3.11 (0.93)
Diastolic blood pressure (mmHg)	79.38 (11.04)
Systolic blood pressure (mmHg)	127.27 (15.28)
Pulse rate (beat/min)	75.62 (15.40)
Cognitive trajectories, *n* (%)	
High	113 (10.10)
Normal	353 (31.55)
Mild	466 (41.64)
Moderate	161 (14.39)
Severe	26 (2.32)
Positive symptoms trajectories, *n* (%)	
Low	788 (70.42)
Moderate	237 (21.18)
High	94 (8.40)
Negative symptoms trajectories, *n* (%)	
Low	828 (73.99)
High-decreased	160 (14.30)
High-increased	131 (11.71)

Abbreviation: SSD, schizophrenia spectrum disorders.

### Pairwise comparisons of cardiometabolic biomarkers

**Figure 1.**
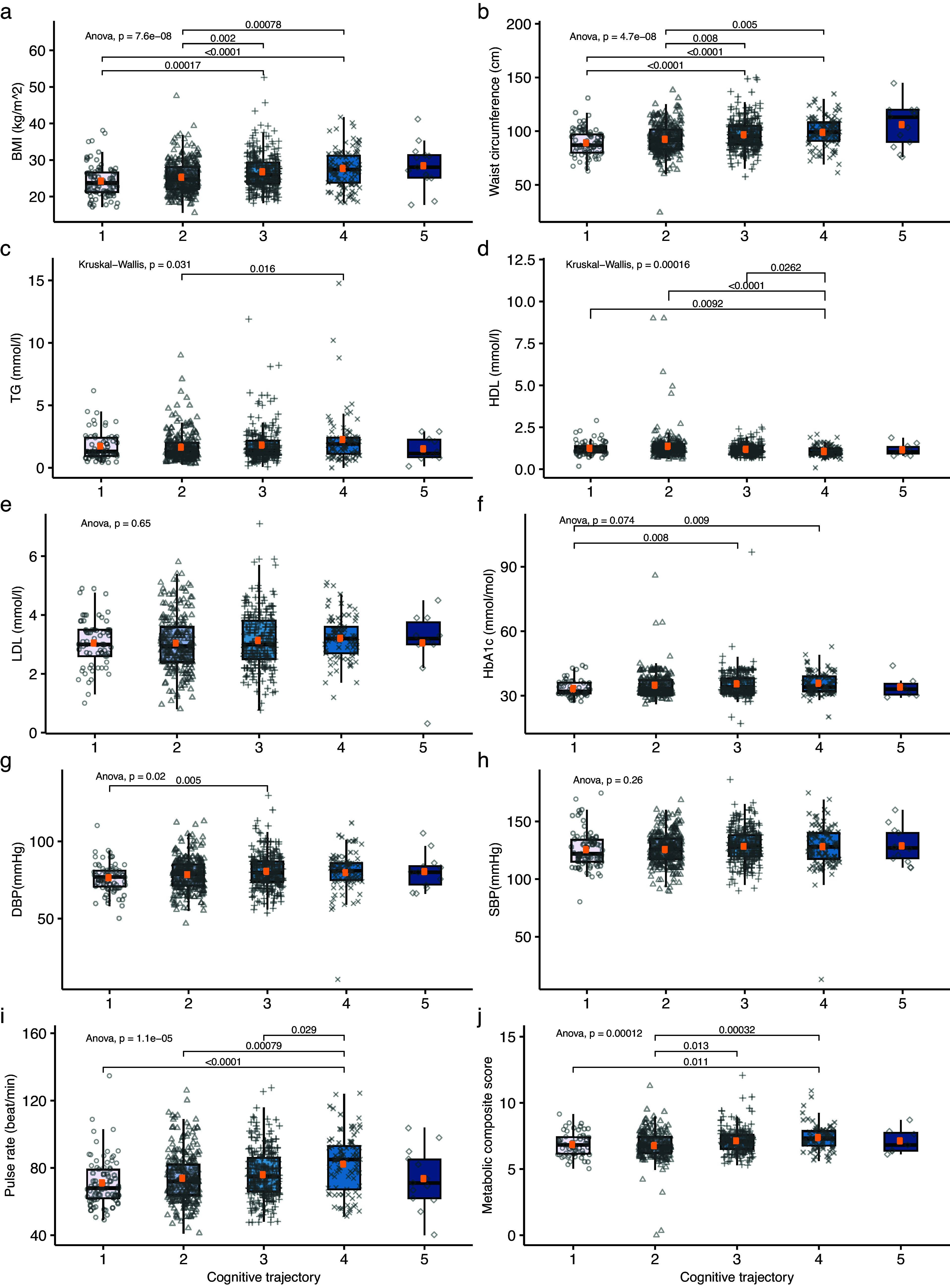
The cardiometabolic profiles of cognitive trajectories (coding represents 1, high; 2, normal; 3, mild; 4, moderate; 5, severe cognitive trajectory).

Patients with mild to severe cognitive impairment had higher BMI (mean_mild_ = 26.78 kg/m^2^, mean_moderate_ = 27.69 kg/m^2^, *P* = 7.6e-08), waist circumference (mean_mild_ = 97.00 cm, mean_moderate_ = 89.50 cm, *P* = 4.7e-08), TG (mean_moderate_ = 2.25 mmol/mol, *P* = 0.031), DBP (mean_mild_ = 80.82 mmHg, *P* = 0.02), pulse rate (mean_mild_ = 76.22 beats/min, mean_moderate_ = 82.30 beats/min, *P* = 1.1e-05) and metabolic composite score (mean_moderate_ = 7.40, *P* = 0.00012) and lower HDL cholesterol (mean = 1.07 mmol/mol, *P* = 0.00016) compared to patients with “High” and “Normal” cognitive function (Figure 1).

**Figure 2.**
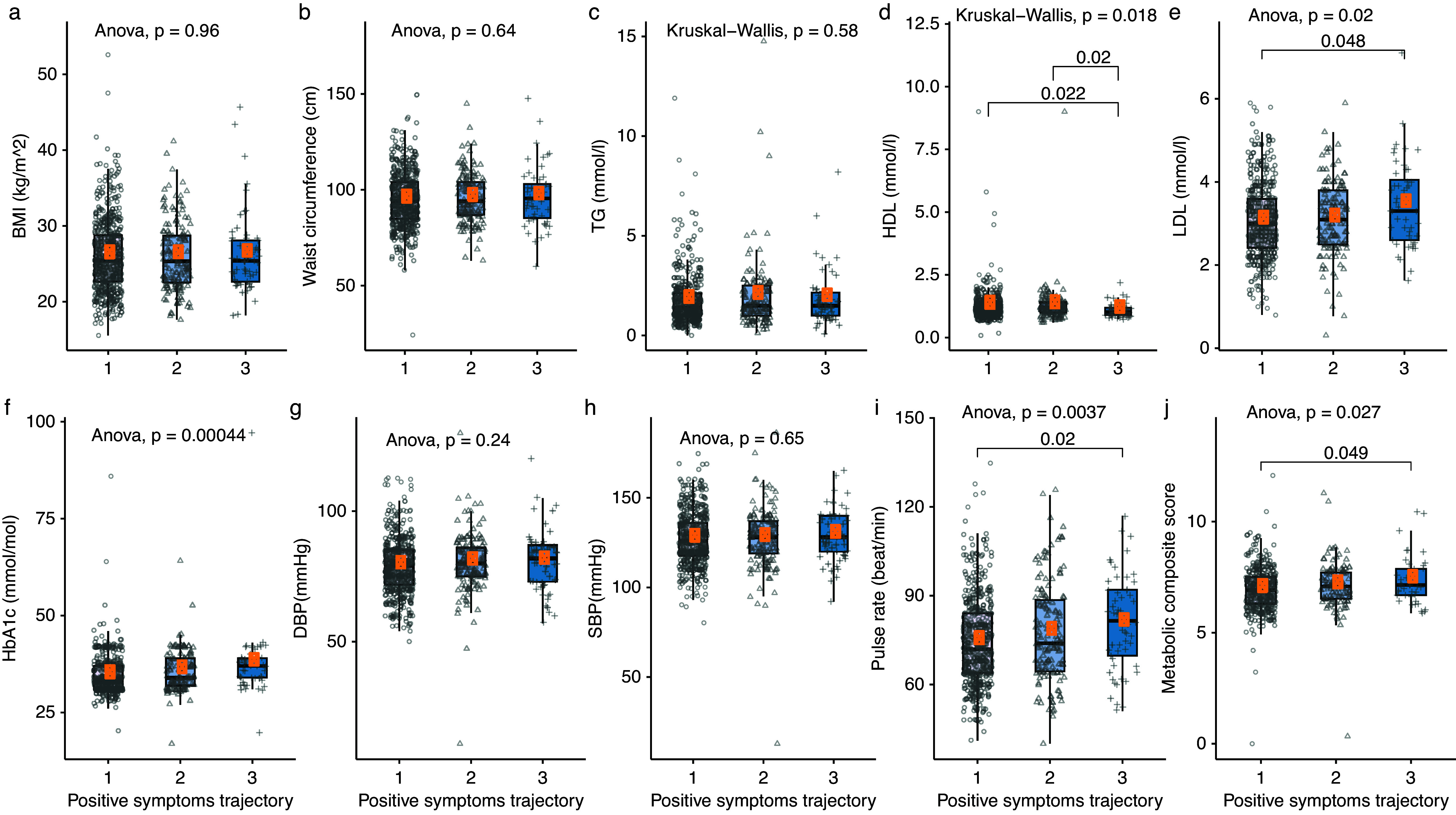
The cardiometabolic profiles of positive symptoms trajectories (coding represents 1, low; 2, moderate; 3, high positive symptoms trajectory).

**Figure 3.**
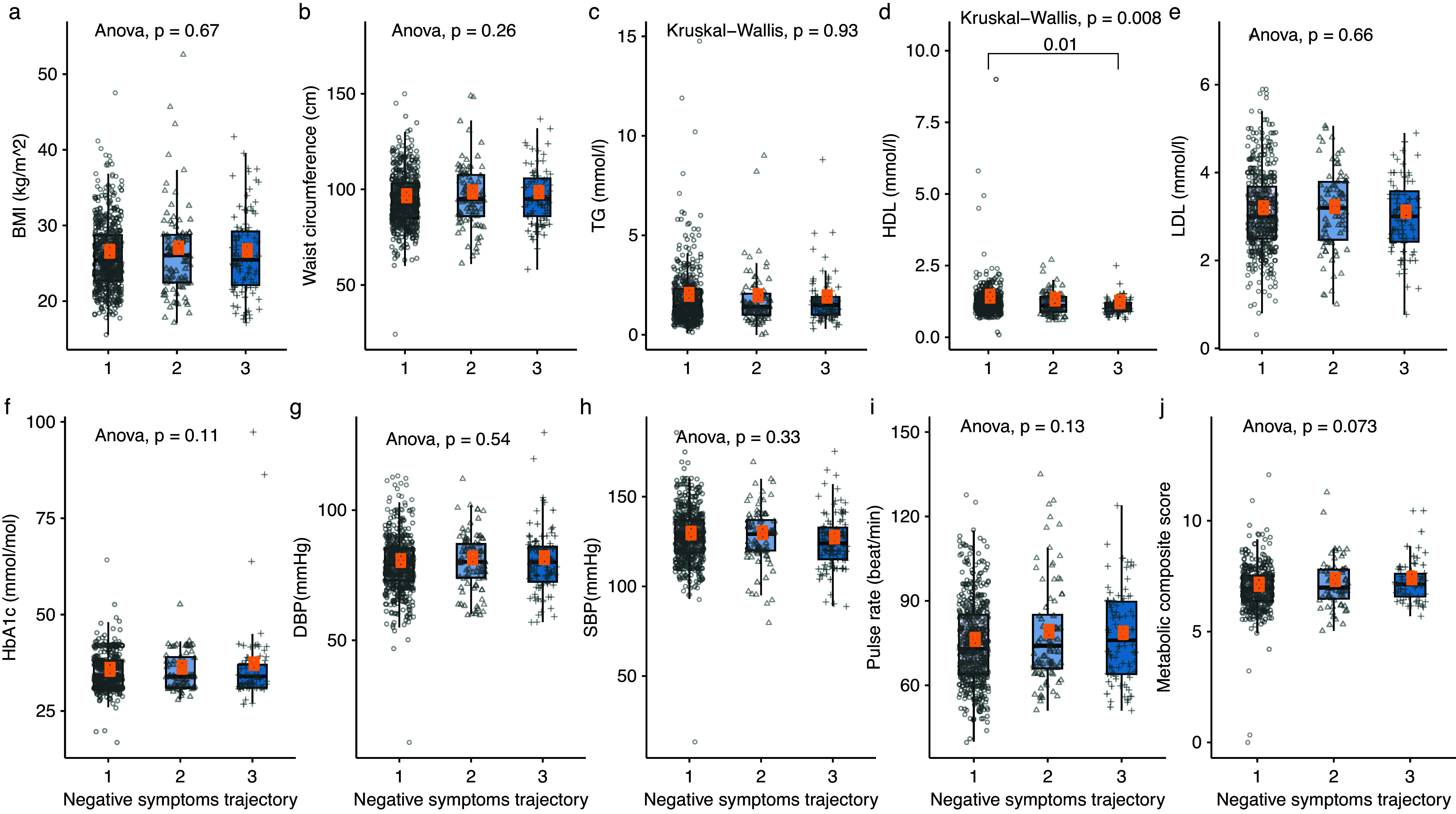
The cardiometabolic profiles of negative symptoms trajectories (coding represents 1, low; 2, high-decreased; 3, high-increased negative symptoms trajectory).

Patients with severe positive symptoms had a significantly higher LDL level (mean_high_ = 3.46 mmol/mol, *P* = 0.02), pulse rate (mean_high_ = 80.68 beats/min, *P* = 0.0037) and metabolic composite score (mean_high_ = 7.37, *P* = 0.027), and lower HDL (mean_high_ = 1.09 mmol/mol, *P* = 0.018) compared with those with “low” severity positive symptoms (Figure 2). The mean HDL levels (mean_high-increased_ = 1.10 mmol/mol, *P* = 0.008) of patients with more severe negative symptoms were significantly lower than that in “low” severity subgroup (Figure 3).

### Cognitive trajectories and cardiometabolic biomarkers

Cognitive impairment was significantly associated with increased BMI (mean difference [β], β_high_ = −1.24, 95% CI = –2.28 to 0.20, *P* = 0.02), TG (β_moderate_ = 0.54, 95% CI = 0.17 to 0.92, *P* < 0.001), and SBP (β_mild_ = 2.74, 95% CI = 0.11 to 5.37, *P* = 0.04, Table 2A). No significant associations were observed in multinomial regression of cognitive trajectories (Table 3A).

**Table 2.** Linear association of cardiometabolic biomarkers over clinical trajectories

**Predictors** **β (95%CI)**	**BMI** **(N=559)**	**WC** **(N= 532)**	**TG** **(N=474)**	**Reversed HDL** **(N=474)**	**LDL** **(N=467)**	**HbA1c** **(N=456)**	**DBP** **(N=546)**	**SBP** **(N=546)**	**PR** **(N=544)**	**MCS** **(N=404)**
**A. Cognitive trajectory**										
High	-1.24 (-2.28-0.20)^**^	-3.25 (-8.03,1.79)	*Removed*	*Removed*	*Removed*	*Removed*	-2.71 (-5.59,0.17)^⁎^	*Removed*	*Removed*	-0.07(-0.52,0.38)
Normal	-1.54 (-3.28,0.85)*	-2.55 (-5.57,0.46)	*Removed*	-0.11 (-0.24,0.02)	*Removed*	*Removed*	*Removed*	*Removed*	*Removed*	-0.21(-0.48,0.06)
Mild	*Removed*	*Removed*	*Removed*	*Removed*	*Removed*	*Removed*	1.84 (-0.11,3.78) ^⁎^	2.74 (0.11,5.37)^**^	1.36(-1.93,4.65)	*Removed*
Moderate	*Removed*	*Removed*	0.54 (0.17,0.92)^***^	*Removed*	*Removed*	*Removed*	*Removed*	*Removed*	4.31(-0.54,9.15)^⁎^	*Removed*
Severe	*Removed*	*Removed*	*Removed*	*Removed*	*Removed*	*Removed*	*Removed*	*Removed*	*Removed*	*Removed*
**B. Positive symptoms trajectory**										
Low	*Removed*	*Removed*	*Removed*	*Removed*	*Removed*	-2.01(-3.21, -0.82)^**^	*Removed*	*Removed*	-2.25(-5.17, -0.67)	*Removed*
Moderate	*Removed*	*Removed*	*Removed*	*Removed*	*Removed*	*Removed*	*Removed*	*Removed*	*Removed*	*Removed*
High	*Removed*	*Removed*	*Removed*	*Removed*	*Removed*	*Removed*	*Removed*	*Removed*	*Removed*	*Removed*
**C. Negative symptoms trajectory**										
Low	*Removed*	*Removed*	*Removed*	-0.11 (-0.24,0.02)	*Removed*	*Removed*	*Removed*	*Removed*	*Removed*	-0.20(-0.43,0.03)^⁎^
High-Decreased	*Removed*	*Removed*	*Removed*	*Removed*	*Removed*	*Removed*	*Removed*	*Removed*	*Removed*	*Removed*
High-Increased	*Removed*	*Removed*	*Removed*	*Removed*	*Removed*	*Removed*	*Removed*	-3.92(-7.66,-0.17)^**^	*Removed*	*Removed*
**D. Covariates**										
Age	0.02 (-0.04, 0.08)	0.21 (0.03, 0.38)^**^	0.01 (-0.01, 0.03)	0.01 (0.00, 0.02)*	0.03 (0.02, 0.04)^***^	0.22 (0.14, 0.30)^***^	0.17 (0.05, 0.29)^**^	0.14 (-0.03, 0.31)	-0.25(-0.44,-0.05)^**^	0.03(0.01,0.04)^***^
Gender (Female)	0.30 (-0.32, 1.23)	-4.56 (-7.19, -1.92)^***^	-0.55 (-0.87, 0.22)^***^	-0.26 (-0.40, -0.11)^***^	-0.08 (-0.28, 0.12)	-1.69 (-3.02, -0.36)^**^	-0.99 (-3.14, 1.17)	-9.41 (-12.39, -6.42)^***^	1.42(-1.63,4.47)	-0.62(-0.86,-0.39)^***^
Ethnicity (Caucasian)	*Removed*	*Removed*	*Removed*	*Removed*	*Removed*	*Removed*	*Removed*	*Removed*	*Removed*	*Removed*
IQ	-0.03 (-0.06,0.00)*	-0.12(-0.21,-0.02)^**^	*Removed*	*Removed*	*Removed*	*Removed*	*Removed*	*Removed*	-0.09 (-0.19, 0.01)^⁎^	-0.01 (-0.02, 0.00)^⁎^
Illness duration	0.19 (0.10, 0.29)^***^	0.37(0.10, 0.65)^**^	*Removed*	*Removed*	*Removed*	*Removed*	*Removed*	*Removed*	0.50(0.18,0.81)^**^	*Removed*
Education	*Removed*	*Removed*	*Removed*	*Removed*	*Removed*	*Removed*	-0.27 (-0.50, -0.04)^**^	*Removed*	*Removed*	*Removed*
Cigarettes use	*Removed*	*Removed*	*Removed*	*Removed*	*Removed*	*Removed*	*Removed*	*Removed*	0.10(0.02,0.19)^**^	*Removed*
Alcohol use	*Removed*	*Removed*	*Removed*	*Removed*	0.01 (0.00, 0.01)*	*Removed*	0.08(-0.00,0.16) ^⁎^	*Removed*	*Removed*	*Removed*

Abbreviations: β: effect size; CI: Confidence Interval; BMI: body mass index; WC: waist circumference; TG: Triglycerides; HDL: Reversed High density lipoprotein; LDL: Low density lipoprotein; HbA1c: Glycated haemoglobin; DBP: Diastolic blood pressure; SBP: Systolic blood pressure; PR: Pulse rate; MCS: Metabolic composite score

Removed: The variable was excluded from the final model

N: sample size of the model fitting

Significance level: ^***^: *P*-value < 0.001; ^**^: *P*-value < 0.05; ^⁎^: *P*-value < 0.1

**Table 3.** The association of clinical trajectories over cardiometabolic biomarkers

Predictors OR (95% CI)	A. Cognitive trajectories			
	Normal (*N* = 150)	Mild (*N* = 136)	Moderate (*N* = 55)	Severe (*N* = 6)
Age	1.07 (0.99, 1.15)	1.14 (1.04, 1.25)**	1.17 (1.06,1.30)**	1.20 (1.02, 1.42)**
Gender (Female)	0.92 (0.34, 2.50)	1.02 (0.32, 3.25)	1.86 (0.44, 7.91)	2.14 (0.15, 31.52)
IQ	0.90 (0.87, 0.93)***	0.79 (0.76, 0.83)***	0.69 (0.64, 0.73)***	0.58 (0.47,0.70)***
Illness duration	1.02 (0.93, 1.12)	0.98 (0.87, 1.10)	1.09 (0.94, 1.26)	0.89 (0.62, 1.28)
Cigarettes use	1.02 (0.99, 1.06)	1.01 (0.97, 1.05)	1.03 (0.98, 1.08)	0.97 (0.87, 1.09)
HbA1c	1.07 (0.96,1.18)	1.11 (0.99, 1.24)***	1.13 (0.99, 1.28)***	0.97 (0.76, 1.23)

Predictors OR (95% CI)	B. Positive symptom trajectories		C. Negative symptom trajectories	
	Moderate (*N* = 82)	High (*N* = 31)	High-Decreased (*N* = 51)	High-Increased (*N* = 54)
Age	0.99 (0.95, 1.03)	0.99 (0.93, 1.06)	0.99 (0.95, 1.04)	1.02 (0.98, 1.07)
Gender (Female)	1.03 (0.56, 1.90)	10.66 (1.37, 83.24)**	1.35 (0.61, 2.97)	1.84 (0.75, 4.50)
IQ	0.99 (0.97, 1.00)***	0.97 (0.94, 0.99)**	1.00 (0.98, 1.02)	0.97 (0.95, 0.99)**
Illness duration	1.02 (0.95, 1.09)	1.13 (1.03, 1.23)**	N/A	N/A
Cigarettes use	1.02 (1.00, 1.10)***	1.03 (1.01, 1.06)**	N/A	N/A
HbA1c	1.05 (1.01, 1.10)**	1.08 (1.02, 1.14)**	N/A	N/A
WC	0.99 (0.97, 1.01)	0.97 (0.94, 1.01)	N/A	N/A
Alcohol use	N/A	N/A	0.99 (0.96, 1.02)	0.94 (0.89, 0.99)**
BMI	N/A	N/A	1.04 (0.97, 1.12)	0.93 (0.86, 1.01)
Reversed HDL	N/A	N/A	1.33 (0.68, 2.62)	6.25 (1.81, 21.59)**
DBP	N/A	N/A	1.04 (1.01, 1.08)**	1.04 (1.00, 1.08)**
SBP	N/A	N/A	0.97 (0.94, 1.00)**	0.97 (0.94, 1.00)

Abbreviations: BMI, body mass index; CI, confidence interval; DBP, diastolic blood pressure; HDL, high-density lipoprotein; HbA1c, glycated hemoglobin; N, sample size of the model fitting; OR, odds ratio; SBP, systolic blood pressure; WC, waist circumference.

*Note:* Reference category of cognitive trajectories: high-performance (n = 46); reference category of positive symptoms trajectories: low (n = 280); reference category of negative symptoms trajectories: low (n = 288).

Significance level: ^***^P-value <0.001; ^**^P-value <0.05; *P-value <0.1.

**Table 4.** The mediated effect of antipsychotics in the relationship of cognitive trajectories and cardiometabolic biomarkers

Predictors β (95% CI)	Total effect	Direct effect	Indirect effect	A-path effect
BMI	1.19 (0.78, 1.61)***	1.19 (0.78, 1.61)***	−0.00(−0.02, 0.02)	0.00 (−0.06, 0.07)
WC	1.79 (−0.22, 3.80)*	1.79 (−0.23, 3.81)*	−0.01 (−0.21, 0.17)	−0.09 (−0.20, 0.02)
TG	0.10 (−0.14, 0.35)	0.10 (−0.15, 0.34	0.01 (−0.02, 0.04)	−0.09(−0.20, 0.02)*
Reversed HDL	−0.03 (−0.15, 0.09)	−0.03 (−0.16, 0.09)	0.00 (−0.01, 0.02)	−0.10 (−0.22, 0.01)
LDL	−0.01 (−0.10, 0.08)	−0.01 (−0.10, 0.08)	−0.00 (−0.01, 0.01)	0.01 (−0.06, 0.08)
HbA1c	0.30 (−0.22, 0.891)	0.29 (−0.22, 0.80)	0.01 (−0.02, 0.05)	−0.02 (−0.10, 0.05)
DBP	−0.03 (−1.64, 1.59)	−0.04 (−1.66, 1.58)	0.01 (−0.11, 0.15)	−0.06 (−0.17, 0.04)
SBP	1.21 (−0.11, 2.52)*	1.22 (−0.09, 2.64)	−0.01 (−0.11, 0.05)	0.02 (−0.04, 0.09)
PR	1.64 (−0.59, 3.86)	1.47 (−0.76, 3.69)	0.17 (−0.06, 0.53)	−0.08 (−0.19, 0.02)
MCS	0.05 (−0.13, 0.23)	0.04 (−0.14, 0.22)	0.01 (−0.01, 0.04)	−0.10 (−0.23, 0.02)*

Abbreviations: β, effect size; CI, confidence interval; BMI, body mass index; WC, waist circumference; TG, triglycerides; HDL, high-density lipoprotein; LDL, low-density lipoprotein; HbA1c, glycated hemoglobin; DBP, diastolic blood pressure; SBP, systolic blood pressure; PR, pulse rate; MCS, metabolic composite score.

Significance level: ^***^P-value <0.001; ^**^P-value <0.05; *P-value <0.1.

### Positive symptoms trajectories and cardiometabolic biomarkers

Increased HbA1c levels were associated with severity of positive symptoms in both linear (β_low_ = −2.01, 95% CI = −3.21 to −0.82, *P* = 0.001, Table 2B) and multinomial (OR_moderate_ = 1.05, 95% CI = 1.01 to 1.10, *P* = 0.024; OR_high_ = 1.08, 95% CI = 1.02 to 1.14, *P* = 0.006, Table 3B) regression.

### Negative symptoms trajectories and cardiometabolic biomarkers

Cardiometabolic outcomes were not associated with negative symptoms in the linear model (Table 2C). In the multinomial model, more severe negative symptoms were associated with increased DBP (OR_high-decreaced_ = 1.04, 95% CI = 1.01 to 1.08, *P* = 0.02; OR_high-increaced_ = 1.04, 95% CI = 1.00 to 1.08, *P* = 0.048) and decreased HDL (OR_high-increased_ = 6.25, 95% CI = 1.81 to 21.59, *P* = 0.004, Table 3C).

### Mediation analysis for antipsychotics

**Figure 4.**
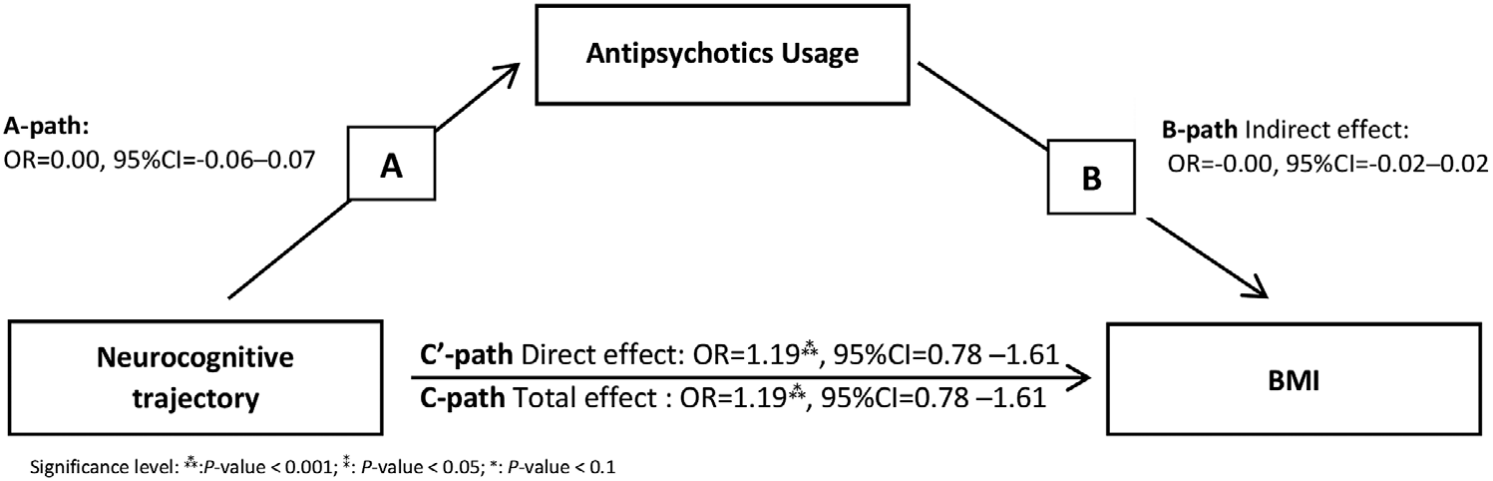
The path model of mediation analysis, e.g., BMI. See also Table 4.

The direct effect (β_total_ = 1.19, 95% CI = 0.78 to 1.61, *P* < 0.001) and total effect (β_direct_ = 1.19, 95% CI = 0.78 to 1.61, *P* < 0.001) of cognitive performance on BMI were significant. Antipsychotic use was neither related to cognitive performance (A-path effect, *P >* 0.05) nor cardiometabolic parameters (indirect effect, bootstrapped CI contained zero). Nondirect or indirect significant effects were observed on other cardiometabolic components, and metabolic composite score (Figure 4 and Table 4).

### Sensitivity analysis

Outliers had low effect on cardiometabolic outcomes. Specifically, there were still significant associations between cognitive impairment and TG (β_moderate_ = 0.34, 95% CI = 0.05 to 0.64, *P* = 0.02), DBP (β_high_ = −4.26, 95% CI = −7.02 to −1.51, *P* = 0.002), and SBP (β_high_ = −5.00, 95% CI = −8.74 to −1.26, *P* = 0.009; β_high_ = −3.85, 95% CI = 6.33 to −0.57, *P* = 0.002). However, the association with BMI disappeared. Decreased HbA1c was associated with more severe negative symptoms (β_high-decreased_ = −1.23, 95% CI = −2.44 to −0.01, *P* = 0.049, Supplementary Table S1).

## Discussion

We investigated the association between cardiometabolic biomarkers, metabolic composite score, and cognitive, positive, negative, and symptoms trajectories in patients with SSD. We found that increased cognitive impairment was significantly associated with increased BMI, TG, and SBP. The higher severity of positive symptoms was also associated with increased HbA1c. We also found that increased DBP and decreased HDL cholesterol were associated with increased severity of negative symptoms. We found no mediation effect for antipsychotics.

### Cognitive impairment and BMI

We found a significant association between cognitive inefficiencies and increased BMI, in line with previous findings in the Chinese [47] and Japanese [48] populations. On the other hand, several factors could affect the BMI level in patients with SSD. First, people with reduced cognitive function are more likely to become overweight or obese because of a decline in executive function [49], which will lead to less frequent energy maintain behaviors like self-monitoring [50]. Besides, a “selfish brain” theory [51, 52] has been put forward and discussed by researchers, stating that cognitive impairment would contribute to an inefficient regulation of brain energy which increases the risk of metabolic dysfunctions.

### Cognitive impairment and blood pressure

We found that cognitive impairment was associated with increased SBP, while both increased diastolic and systolic blood pressure were not associated with cognitive impairment. Our results were partly in line with previous evidence which suggested high blood pressure [53, 54]may cause disruption of the blood–brain barrier and lead to structural abnormalities in blood vessels. The micro- and macro-cerebrovascular alteration and diminished blood flow may eventually lead to memory impairment and other cognitive dysfunction [55].

### Cognitive impairment and dyslipidemia

The relationship between cognitive impairment and dyslipidemia remains controversial. We observed an association between cognitive impairment and TG, but no significant relation was found between HDL and LDL. Liu et al. [56] found the association between lipid parameters and cognitive impairment was heterogeneous in age and gender subgroups. On the contrary, the nonsignificant result was reported by a recent systematic review that cognitive function in all domains did not differ in SSD with or without dyslipidemia [57].

### Cognitive impairment and HbA1c

There was no association between HbA1c and cognitive impairment. Previous studies have suggested a correlation between HbA1c level and poor cognition in recent onset psychosis patients [58, 59]. Most of the previous results were found in patients with diabetes or in elderly population [60, 61], and with differences in race among the included subjects. Chronic hyperglycemia decreases glucose transfer through the blood–brain barriers, resulting in the loss of acetylcholine [62, 63] and dysregulation of cortical neurons [64]. Most patients in our samples who had a recent psychosis onset did not exceed the HbA1c threshold of diabetes, which seems to suggest that the association between glucose level and cognitive dysfunction may only be present in patients with a longer illness duration and more severe hyperglycemia.

### Positive and negative symptoms and cardiometabolic biomarkers

Our study indicated that increased positive symptom severity was associated with increased HbA1c levels, while negative symptom severity was related to DBP and HDL cholesterol. The relationship between cardiometabolic parameters and positive and negative symptoms remains inconclusive. Chen et al.’s study [65] suggested negative association between insulin resistance and positive symptoms in Chinese SSD patients, though no correlation was found with negative symptoms. Wedervang-Resell et al. [66] found a correlation between higher PANSS negative score and elevated TG levels. Conversely, the severity of negative symptoms exhibited an inverse association with BMI and TG levels, and a positive association with HDL levels, while no correlation was observed between positive symptoms and cardiometabolic parameters [67, 68]. Consequently, further investigation is needed to determine whether the severity of positive and negative symptoms influences the risk of developing cardiometabolic outcomes.

### Antipsychotics and cardiometabolic biomarkers

We found the association between cognitive symptoms and cardiometabolic parameters is independent of the use of antipsychotics. The side effect of second-generation antipsychotics is often seen as an important factor to develop cardiometabolic outcomes [69]. For example, Gupta et al. found atypical antipsychotics were related to glucose dysregulation or diabetes mellitus [70]; Melkersson et al. observed elevated levels of insulin and blood lipids in patients treated with olanzapine [71]. However, we did not observe an indirect effect for the use of antipsychotics on cardiometabolic biomarkers in our samples. This may be ascribed to the antipsychotic medication switch in clinical practice based on the appearance of adverse effects [72] like weight gain, which has not been captured at follow-up point. An alternative explanation could be that patients who exhibit poor medication compliance might experience less antipsychotic-induced cardiometabolic disorders.

### Strengths and limitations

This comprehensive study had a large sample of patients with SSD. The assessments of these symptoms were also comprehensive, which contributes to the accurate estimate of long-term cognitive trajectories and psychotic symptoms trajectories. Along with these advantages, this is a cross-sectional analysis, which hampers evaluation of the causal effect of cognitive impairment on metabolic outcomes or vice versa. Secondly, the mean age of our samples is young, which can lead to underestimate the effect of cardiometabolic multimorbidity in subjects. Thirdly, while our study focused on the primary factors of interest, it’s worth noting several factors like diet, exercise, and concomitant medications that might impact the cardiometabolic outcomes, were not taken into our analysis. Although their effect could be less likely to bias the estimates of associations, it may potentially neglect the assessment of studying interactions. Finally, our study followed up to 6 years. However, considering the long-term nature of clinical symptoms and cardiometabolic dysregulations in patients with SSD, for example, cardiometabolic dysregulations could be associated with the decline of cognitive function over 6 years in middle-aged [73] and old [74] populations, it would be an important avenue for future research.

### Clinical and public health implementations

The current findings emphasize the need for regular monitoring and screening of cardiometabolic risk biomarkers in patients with SSD. Earlier interventions such as dosage adjustment or switching to different antipsychotics with a lower metabolic risk if necessary, would help to decrease the risk of developing cardiovascular diseases in their later life.

## Conclusion

We demonstrated the association between BMI, TG and SBP and cognitive impairment, and between elevated levels of HbA1c, HDL cholesterol, and DBP with positive and negative symptoms in patients with SSD. The results suggested poorer cardiometabolic parameters are associated with both worse cognitive function and more severe schizophrenia symptoms. The observed associations underscore the need for early identification of patients with SSD at risk of cardiometabolic outcomes. Future studies would investigate patients with a wider age range and severity of metabolic complications to elucidate the underlying causality of the observed associations. For instance, studies have highlighted that inflammation is a shared characteristic of both cardiometabolic disorders and psychosis [75]. Therefore, it is advisable to include inflammatory biomarkers, such as C-reactive protein, and pro-inflammatory cytokines like interleukin-6, for a more comprehensive exploration.

## Supporting information

Zhao et al. supplementary material Zhao et al. supplementary material

## Supplementary material

The supplementary material for this article can be found at https://doi.org/10.1192/j.eurpsy.2023.2477.
