# Supplementary material for: Association of clinical symptoms and cardiometabolic dysregulations in patients with schizophrenia spectrum disorders
Source: Eur Psychiatry. 2023 Dec 13;67(1):e7. doi: 10.1192/j.eurpsy.2023.2477 (PMC10964276; doi:10.1192/j.eurpsy.2023.2477)
Supplement: Zhao et al. supplementary material [file S092493382302477Xsup001.docx]

**Supplementary Materials**

**Association of Clinical Symptoms and Cardiometabolic Dysregulations in Patients with Schizophrenia Spectrum Disorders**

Chenxu Zhao^1^ et.al, 2023

| **Predictors**  **β (95%CI)** | **BMI**  **(N=554)** | **WC**  **(N= 530)** | **TG**  **(N=468)** | **Reversed HDL**  **(N=470)** | **LDL**  **(N=464)** |
| --- | --- | --- | --- | --- | --- |
| **S1A Neurocognitive trajectory** | | | | | |
| High | *Removed* | -3.00 (-7.90, 1.91) | *Removed* | *Removed* | *Removed* |
| Normal | *Removed* | -2.22 (-5.16,0.71) | *Removed* | *Removed* | *Removed* |
| Mild | 0.92(-0.02, 1.85)^⁎^ | *Removed* | *Removed* | *Removed* | *Removed* |
| Moderate | 1.28 (-0.10, 2.66)^⁎^ | *Removed* | 0.34(0.05,0.64) ^⁑^ | 0.04 (-0.06,0.13) | *Removed* |
| Severe | *Removed* | *Removed* | *Removed* | *Removed* | *Removed* |
| **S1B Positive symptoms trajectory** | | | | | |
| Low | *Removed* | *Removed* | *Removed* | *Removed* | *Removed* |
| Moderate | *Removed* | *Removed* | *Removed* | *Removed* | *Removed* |
| High | *Removed* | *Removed* | *Removed* | *Removed* | *Removed* |
| **S1C Negative symptoms trajectory** | | | | | |
| Low | *Removed* | *Removed* | *Removed* | *Removed* | *Removed* |
| High-Decreased | *Removed* | *Removed* | *Removed* | *Removed* | *Removed* |
| High-Increased | *Removed* | *Removed* | *Removed* | 0.06 (-0.02,0.15) | *Removed* |
| **S1D Covariates** | | | | | |
| Age | 0.03 (-0.03, 0.08) | 0.21(0.04 0.37)^⁑^ | 0.01 (-0.01, 0.02) | 0.00 (0.00, 0.01) | 0.03 (0.02, 0.04)^⁂^ |
| Gender (Female) | 0.11 (-0.76, 0.97) | -5.38 (-7.96, -2.81)^⁂^ | -0.42 (-0.66, 0.17)^⁂^ | -0.27 (-0.34, -0.20)^⁂^ | -0.07 (-0.27, 0.12) |
| Ethnicity (Caucasian) | *Removed* | *Removed* | *Removed* | *Removed* | *Removed* |
| IQ | -0.04(-0.07,-0.01)^⁑^ | -0.11(-0.21, -0.02)^⁑^ | *Removed* | *Removed* | *Removed* |
| Illness duration | 0.14(0.04,0.23)^⁑^ | 0.37(0.10,0.64)^⁑^ | *Removed* | *Removed* | *Removed* |
| Education | *Removed* | *Removed* | *Removed* | *Removed* | *Removed* |
| Cigarettes use | *Removed* | *Removed* | *Removed* | *Removed* | *Removed* |
| Alcohol use | *Removed* | *Removed* | *Removed* | *Removed* | 0.01 (0.00, 0.01)^⁎^ |
| Abbreviations: β: effect size; CI: Confidence Interval; BMI: body mass index; WC: waist circumference; TG: Triglycerides; HDL: Reversed High density lipoprotein; LDL: Low density lipoprotein; HbA1c: Glycated haemoglobin; DBP: Diastolic blood pressure; SBP: Systolic blood pressure; PR: Pulse rate; MCS: Metabolic composite score  Removed: The variable was excluded from the final model  N: sample size of the model fitting  Significance level: ^⁂^:*P*-value < 0.001; ^⁑^: *P*-value < 0.05; ^⁎^: *P*-value < 0.1 | | | | | |

**Supplementary Table S1. Sensitivity analysis results of association of cognitive, positive and negative symptoms and cardiometabolic biomarkers (See Table 2)**

| **Predictors**  **β (95%CI)** | **HbA1c**  **(N=451)** | **DBP**  **(N=543)** | **SBP**  **(N=541)** | **PR**  **(N=539)** | **MCS**  **(N=404)** |
| --- | --- | --- | --- | --- | --- |
| **S1A Neurocognitive trajectory** | | | | | |
| High | *Removed* | -4.26 (-7.02,-1.51)^⁑^ | -5.00 (-8.74,-1.26) ^⁑^ | *Removed* | -0.07(-0.52,0.38) |
| Normal | *Removed* | -1.78 (-3.63,0.07)^⁎^ | -3.85 (-6.33,-1.37) ^⁑^ | *Removed* | -0.21(-0.48,0.06) |
| Mild | *Removed* | *Removed* | *Removed* | 0.88 (-2.26,4.02) | *Removed* |
| Moderate | *Removed* | *Removed* | *Removed* | 2.67 (-1.99,7.32) | *Removed* |
| Severe | *Removed* | *Removed* | *Removed* | *Removed* | *Removed* |
| **S1B Positive symptoms trajectory** | | | | | |
| Low | -2.01(-2.91, -1.10)^⁂^ | *Removed* | *Removed* | -2.21(-5.01,0.59) | *Removed* |
| Moderate | *Removed* | *Removed* | *Removed* | *Removed* | *Removed* |
| High | *Removed* | *Removed* | *Removed* | *Removed* | *Removed* |
| **S1C Negative symptoms trajectory** | | | | | |
| Low | *Removed* | *Removed* | *Removed* | *Removed* | -0.20(-0.43,0.03)^⁎^ |
| High-Decreased | *Removed* | *Removed* | *Removed* | *Removed* | *Removed* |
| High-Increased | -1.23(-2.44, -0.01)^⁑^ | *Removed* | -4.92(-8.34,-1.51)^⁑^ | *Removed* | *Removed* |
| **S1D Covariates** | | | | | |
| Age | 0.15 (0.09, 0.21)^⁂^ | 0.15 (0.03, 0.26)^⁑^ | 0.02 (-0.15, 0.19) | -0.22(-0.40,-0.03)^⁑^ | 0.03(0.01,0.04)^⁂^ |
| Gender (Female) | -1.11 (-2.09, -0.13)^⁑^ | -1.27 (-3.31, 0.77) | -8.73 (-11.47, -5.99)^⁂^ | 1.30(-1.61,4.22) | -0.62(-0.86,-0.39)^⁂^ |
| Ethnicity (Caucasian) | *Removed* | *Removed* | *Removed* | *Removed* | *Removed* |
| IQ | *Removed* | *Removed* | *Removed* | -0.12 (-0.22, -0.02)^⁑^ | -0.01 (-0.02, 0.00)^⁎^ |
| Illness duration | *Removed* | *Removed* | 0.24(-0.03,0.52)^⁎^ | 0.43 (0.12, 0.73)^⁑^ | *Removed* |
| Education | *Removed* | -0.23 (-0.44, -0.01)^⁑^ | *Removed* | *Removed* | *Removed* |
| Cigarettes use | *Removed* | *Removed* | *Removed* | 0.08 (0.00, 0.17)^⁎^ | *Removed* |
| Alcohol use | *Removed* | 0.08 (0.00, 0.15)^⁑^ | 0.09(-0.01,0.19)^⁎^ | *Removed* | *Removed* |
| Abbreviations: β: effect size; CI: Confidence Interval; BMI: body mass index; WC: waist circumference; TG: Triglycerides; HDL: Reversed High density lipoprotein; LDL: Low density lipoprotein; HbA1c: Glycated haemoglobin; DBP: Diastolic blood pressure; SBP: Systolic blood pressure; PR: Pulse rate; MCS: Metabolic composite score  Removed: The variable was excluded from the final model  N: sample size of the model fitting  Significance level: ^⁂^:*P*-value < 0.001; ^⁑^: *P*-value < 0.05; ^⁎^: *P*-value < 0.1 | | | | | |

(S1 continued)
